# Pathview: an R/Bioconductor package for pathway-based data integration and visualization

**DOI:** 10.1093/bioinformatics/btt285

**Published:** 2013-06-04

**Authors:** Weijun Luo, Cory Brouwer

**Affiliations:** ^1^Department of Bioinformatics and Genomics, UNC Charlotte, Charlotte, NC 28223 and ^2^UNC Charlotte Department of Bioinformatics and Genomics, North Carolina Research Campus, Kannapolis, NC 28081, USA

## Abstract

**Summary:** Pathview is a novel tool set for pathway-based data integration and visualization. It maps and renders user data on relevant pathway graphs. Users only need to supply their data and specify the target pathway. Pathview automatically downloads the pathway graph data, parses the data file, maps and integrates user data onto the pathway and renders pathway graphs with the mapped data. Although built as a stand-alone program, Pathview may seamlessly integrate with pathway and functional analysis tools for large-scale and fully automated analysis pipelines.

**Availability:** The package is freely available under the GPLv3 license through Bioconductor and R-Forge. It is available at http://bioconductor.org/packages/release/bioc/html/pathview.html and at http://Pathview.r-forge.r-project.org/.

**Contact:** luo_weijun@yahoo.com

**Supplementary information:** Supplementary data are available at *Bioinformatics* online.

## 1 INTRODUCTION

The pathway-based approach has been widely used in high-throughput data analysis (Emmert-Streib and Glazko, 2011; Kelder *et al.*, 2010; Khatri *et al.*, 2012). It has been successfully and routinely applied to gene expression (both microarray and RNA-Seq) (Luo *et al.*, 2009), genetic and GWAS (Wang *et al.*, 2010), proteomic and metabolomics data (Perroud *et al.*, 2006; Xia and Wishart, 2010). Compared with the individual gene/molecule-based approach, pathway analysis is more sensitive, consistent and informative (Luo *et al.*, 2009).

R/Bioconductor has become a primary software environment for high-throughput data analysis and visualization (Gentleman *et al.*, 2004). Numerous pathway analysis methods and data types are implemented in R/Bioconductor, yet there has not been a dedicated and established tool for pathway-based data integration and visualization.

In this article, we introduce a novel package called Pathview. We did a detailed comparison between Pathview and existing pathway tools in R/Bioconductor and other languages in Supplementary Table S2. Pathview provides three features that are rarely implemented well in other tools: (i) fully accessible and functional pathway visualization. It adheres to human readable pathway definitions and layouts like KEGG (Ogata *et al.*, 1999). No previous KEGG base tools provide full graphics, including node/edge attribute modifications, node/edge labels, legends and color keys. (ii) Strong data integration capacity. It integrates and works with data of different types (different omic levels, literature and so forth), IDs, formats, attributes, species and so forth. As far as we know, no other tool provides such extensive data mapping and integration support. (iii) Easy to automate and integrate with pathway analysis tools. Only a few tools can be directly automated and fully integrated into pathway analysis pipelines (Supplementary Table S2: automated analysis column). None of these features are brand new, but surprisingly, few of the existing tools provide satisfactory functionality in these aspects.

## 2 MAIN FEATURES

### 2.1 Overall design

The Pathview package can be divided into four functional modules: Downloader, Parser, Mapper and Viewer, as depicted in Supplementary Figure S1. Most importantly, Pathview maps and renders user data on relevant pathway graphs.

### 2.2 Data visualization

**Fig. 1.**
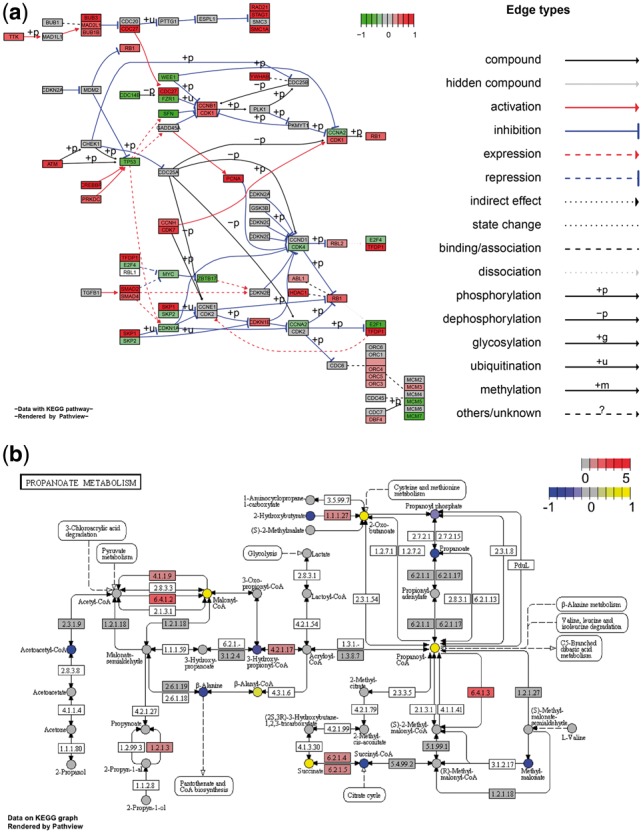
Example Pathview graphs: (**a**) Graphviz view on a canonical signaling pathway (hsa04110 Cell cycle) with gene data only, (**b**) KEGG view on a metabolic pathway (hsa00640 Propanoate metabolism) with both discrete gene data and continuous metabolite data integrated. The same graphs at a higher resolution or with a different color scheme are shown in Supplementary Figures S3 and S4

Pathview generates both native KEGG view (Fig. 1b) and Graphviz (Ellson *et al.*, 2002) view (Fig. 1a) for pathways. Both graph styles adhere to human readable pathway definition and layout, yet still allow proper modification and customization of node and edge attributes. KEGG view retains all pathway meta-data, i.e. spatial and temporal information, tissue/cell types, inputs, outputs and connections. This is important for readability and interpretation of pathway biology. Graphviz view provides better control of node and edge attributes, better view of pathway topology and better understanding of the pathway analysis statistics. The different workflows for these two types of view are merged in Pathview (Supplementary Fig. S1). This keeps the user interface simple and consistent.

### 2.3 Data integration

Pathview provides strong support for data integration (Supplementary Table S1). It can be used to integrate, analyze and visualize a wide variety of biological data: gene expression, protein expression, metabolite level, genetic association, genomic variation, literature record and other data types mappable to pathways. Notably, it can be directly used for metagenomic data when the data are mapped to KEGG ortholog pathways. The integrated Mapper module maps 12 types of gene or protein IDs, and 21 types of compound or metabolite related IDs to standard KEGG gene or compound IDs, and also maps between these external IDs. For other types of IDs (for instance, Affymetrix microarray probe set IDs) not included in the common ID lists, Pathview’s auxiliary functions will map user data to pathways when users provide the ID mapping data manually. Pathview applies to pathways for over 2000 species, and species can be specified in multiple formats: KEGG code, scientific name or common name. In addition, Pathview works with different data attributes and formats, both continuous and discrete data (Fig. 1b and Supplementary Table S1), either in matrix or vector format, with single or multiple samples/experiments and so forth.

### 2.4 Automated and integrated analysis

Pathview is open source, fully automated and error-resistant. Therefore, it seamlessly fits in integrated pathway or gene set analysis workflows.

Pathview can be easily integrated with a wide variety of existing tools in or communicating to R/Bioconductor for high-throughput data analysis and pathway analysis. In the package vignette, we show an integrated analysis using Pathview with another Bioconductor package gage (Luo *et al.*, 2009).

In automated pathway analysis, we frequently use heatmap, scatter plots or stacked line plots to view the perturbation patterns. These plots are simple and can be generated quickly in batches. However, they contain little information beyond the numeric changes. With Pathview, we can view molecular perturbations in intuitive and informative pathway contexts. Importantly, such graphs can be generated equally efficient as the classical scatter or line plots. This will greatly improve the analysis and interpretation of high-throughput molecular data.

KEGG XML data files frequently contain minor deficiencies, inconsistent, incomplete or even error records because of manual curation. These deficiencies adversely affect the parsing, mapping and rendering processes and automation. Pathview accommodates these deficiencies, corrects them or skips the problematic pathway with warning. For example, Pathview Parser corrects for the improper KEGG definition of enzyme-compound interactions by merging and resolving the conflicting ECrel record and associated reactions records (Supplementary Fig. S2). In normal cases, Pathview uses KEGGgraph package (Zhang and Wiemann, 2009) to parse KEGG XML data files.

## 3 DISCUSSION AND CONCLUSION

Pathview maps and renders user data onto pathway graphs, which are intuitive, informative and well annotated. It integrates and works with a large variety of data types, IDs, formats and attributes. Pathview can be easily combined with other tools for automated and efficient pathway analysis pipelines. Currently, Pathview works with all types and species of KEGG pathways. We plan to support pathways from Reactome (Croft *et al.*, 2011), NCI Pathway Interaction and other databases based on needs in the future.

*Funding*: UNC general administration (to W.L. and C.B.).

*Conflict of Interest*: none declared.

## Supplementary Material

Supplementary Data
