# Supplementary material for: Pathview: an R/Bioconductor package for pathway-based data integration and visualization
Source: Bioinformatics. 2013 Jun 4;29(14):1830–1. doi: 10.1093/bioinformatics/btt285 (PMC3702256; doi:10.1093/bioinformatics/btt285)
Supplement: Supplementary Data [file supp_29_14_1830__index.html]

Pathview: an R/Bioconductor package for pathway-based data integration and visualization — Pathview: an R/Bioconductor package for pathway-based data integration and visualization — Supplementary Data 

# Pathview: an R/Bioconductor package for pathway-based data integration and visualization

## 

files

**Files in this Data Supplement:**

- Supplementary Data - docx file
