# Supplementary material for: Pathview: an R/Bioconductor package for pathway-based data integration and visualization
Source: Bioinformatics. 2013 Jun 4;29(14):1830–1. doi: 10.1093/bioinformatics/btt285 (PMC3702256; doi:10.1093/bioinformatics/btt285)
Supplement: Supplementary Data [file supp_btt285_pathview_suppleTabFig_2_updated.docx]

**Supplementary Table S1**. The current data integration capacity of Pathview.

| **Data Attribute** | **Pathview Coverage** |
| --- | --- |
| Type | Gene, protein, metabolite, genetics, literature, and others |
| ID | 12 gene IDs, 21 compound IDs |
| Species | 2299 KEGG species and ortholog |
| Value | Continuous, discrete |
| Format | Matrix, vector |

**Supplementary Table S2**. Comparison between Pathview and other publicly available pathway processing and visualization tools.

Most of these tools were published in Bioinformatics. KEGG based tools are list first, then other tools.

*Full graphic output, including all graphic components in output graphs: i.e. node/edge attributes and labels, legend, color key(s). “No” means more than 2 components are missing on result graphs. Note that tools like ChiBE display missing elements interactively outside pathway graphs.

#For data mapping, gene data and compound data are general concepts, including genes, transcripts, proteins or compounds, metabolites, drugs etc.

| **Tool** | **Description** | **Pathway Supported** | **Graph Style** | ***Full Graphic Output** | **Data Down-Load** | **#Data Mapping** | **Data Integration** | **Automated Analysis** | **Interface** | **OS** |
| --- | --- | --- | --- | --- | --- | --- | --- | --- | --- | --- |
| Pathview | Data mapping, integration and visualization | KEGG (all pathways, species, KO) | KEGG, Graphviz | Yes | Yes | Any data mappable | Strong | Yes | R/Bioconductor | Multiple |
| KGML-ED | Graph editing | KEGG | KEGG | No | No | No | No | No | Java GUI | Multiple |
| KEGGgraph | Parsing, analysis | KEGG | Graphviz | No | Yes | No | No | No | R/Bioconductor | Multiple |
| KEGGtranslator | Format conversion | KEGG | KEGG | No | No | No | No | No | Java GUI and command-line | Multiple |
| KEGGParser | Parsing, editing | KEGG | KEGG | No | Yes | No | No | No | Matlab | Multiple |
| KEGGanim | Data mapping, animation | KEGG (14 organisms) | KEGG, animation | No node labels | No | Gene data | No | No | Web GUI | Multiple |
| KEGGConverter | Pathway fusion, format conversion | KEGG metabolic pathway | No graph | No | No | No | No | No | Java command-line and web GUI | Multiple |
| g-language | Data mapping | KEGG (predefined subset) | KEGG | No color key | No | Gene and compound data | Limited | No | Web GUI | Multiple |
| VisANT | Visualization, editing, prediction and construction | KEGG | Compound graphs | No | Yes | Gene data | No | Yes | Java GUI and command-line | Multiple |
| ChiBE | Pathway editing, visualization | BioPAX, basic SIF format | Compound graphs, SBGN | No | Yes | Gene and compound data | Limited | No | Java GUI | Multiple |
| Cytoscape and plugins | Network integration, analysis, and visualization | Pathway and molecular interaction | Various layout algorithms | Yes | Yes | Molecular data | Strong | Limited | Java platform and GUI | Multiple |
| GenMAPP | Data mapping, graph editing | Community curated | Manual layout | Yes | No | Gene data | Limited | No | Visual Basic GUI | Windows |


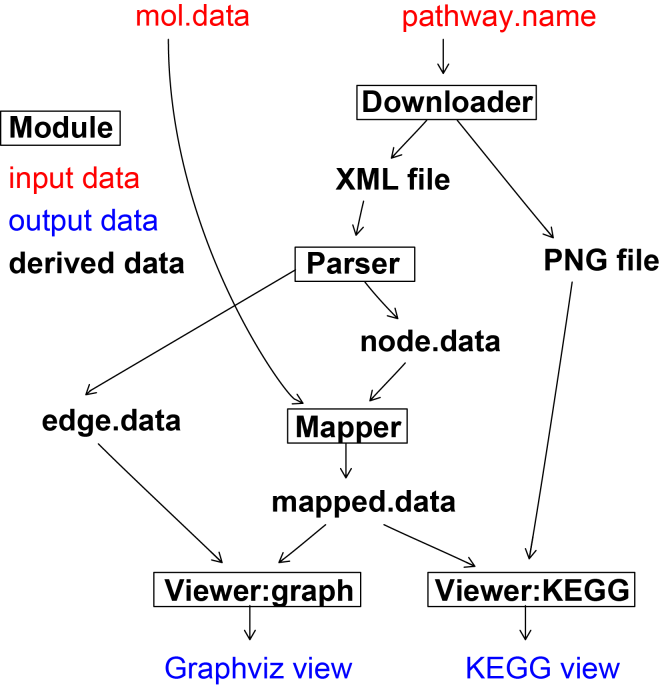


**Supplementary Figure S1**. Pathview workflow with KEGG pathways.


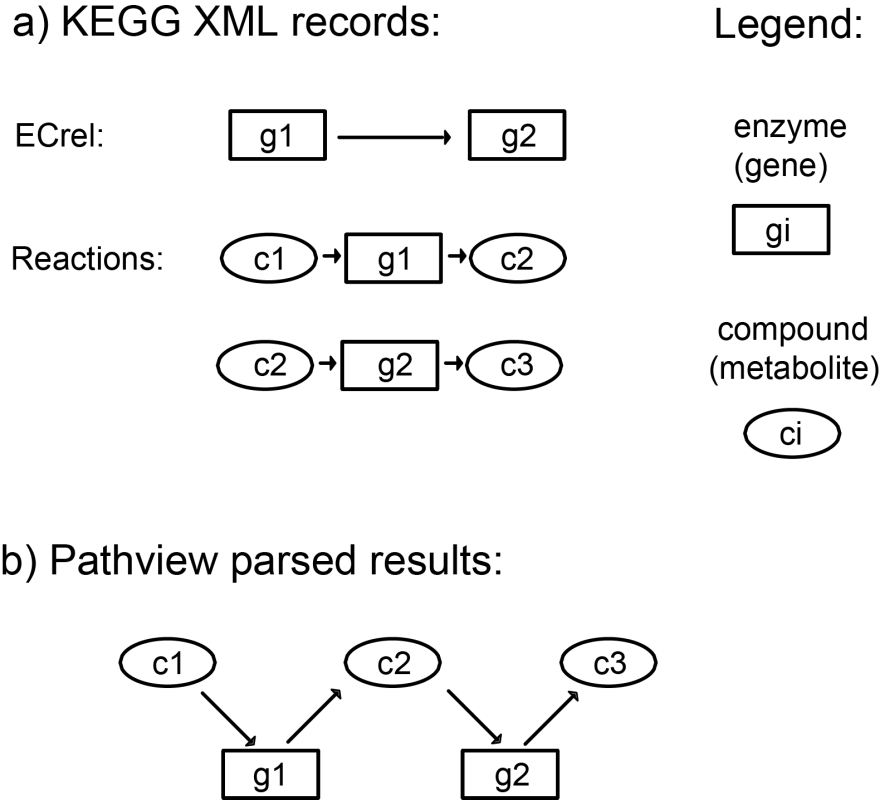


**Supplementary Figure S2**. Pathview Parser corrects for the improper KEGG definition of enzyme-compound interactions: a) In KEGG XML files, enzyme-compound interactions are defined as two separate yet redundant types of records: the ECrel (enzyme-enzyme relation) and associated Reactions. Note the ECrel record is flawed and conflicts with the Reaction records because there is actually no direct interaction between g1 and g2. Therefore, it is improper to parse ECrel records directly into interactrions (or edges) in pathway graphs and skip the Reaction records as have done by other KEGG parsing programs; b) Pathview merges these records and converts them to consecutive interactions between substrate compound(s), gene (enzyme) and product compound(s).


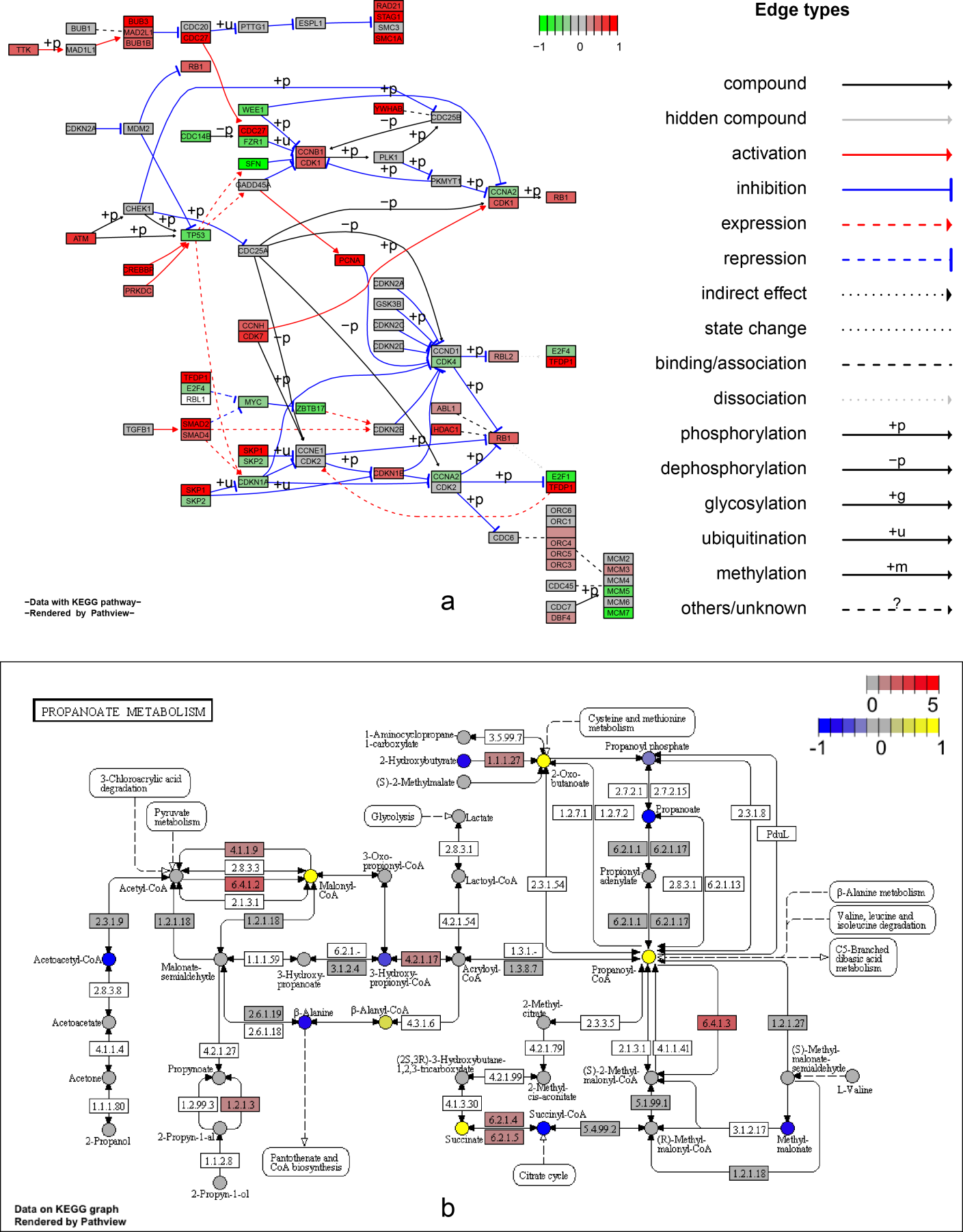


**Supplementary Figure S3.** Example Pathview graphs at a higher resolution: a) Graphviz view on a canonical signaling pathway (hsa04110 Cell cycle) with gene data only, b) KEGG view on a metabolic pathway (hsa00640 Propanoate metabolism) with both discrete gene data and continuous metabolite data integrated.


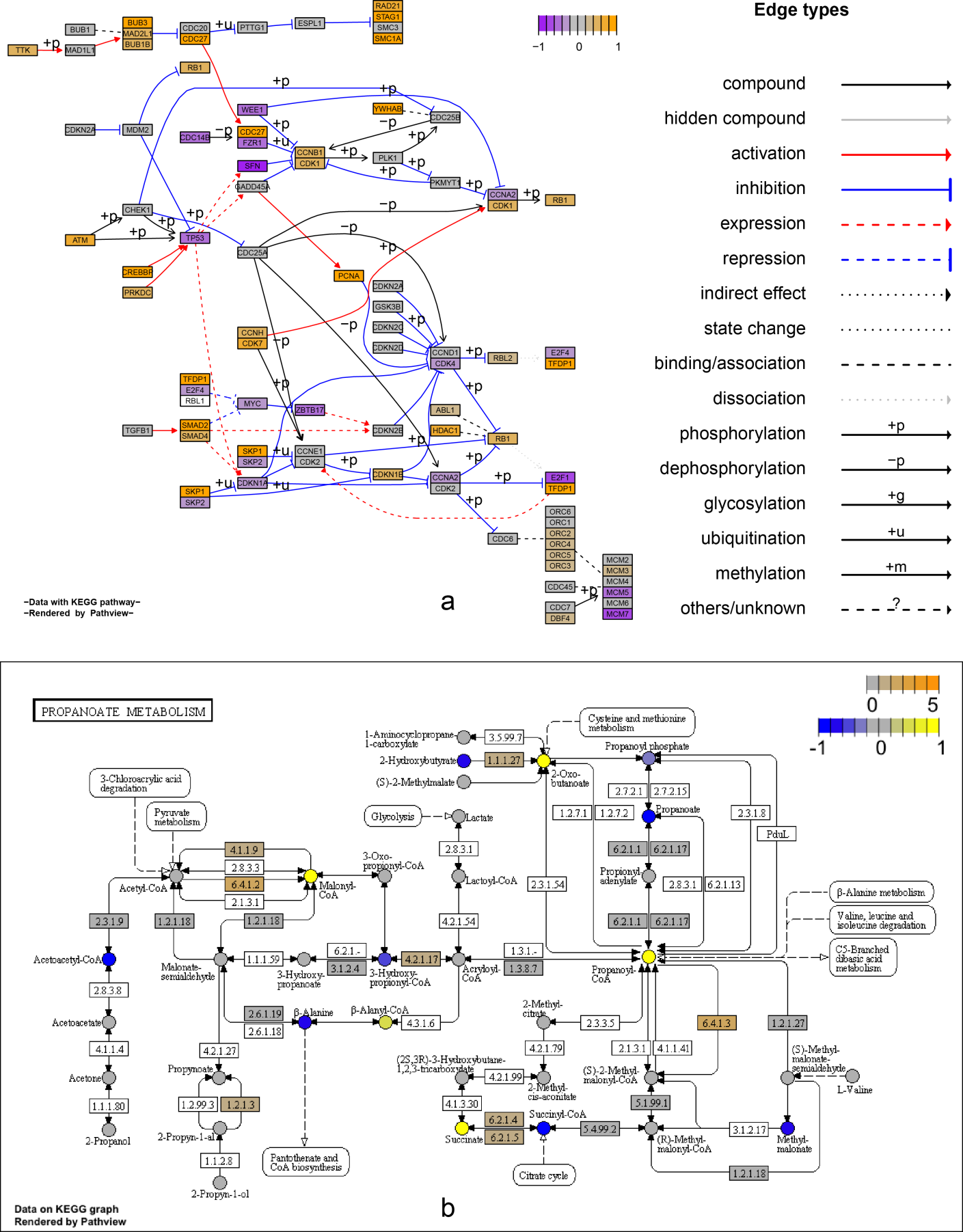


**Supplementary Figure S4.** Example Pathview graphs at a higher resolution with another color scheme: a) Graphviz view on a canonical signaling pathway (hsa04110 Cell cycle) with gene data only, b) KEGG view on a metabolic pathway (hsa00640 Propanoate metabolism) with both discrete gene data and continuous metabolite data integrated.
